# The prevalence and associated clinical correlates of hyperuricemia in patients with bipolar disorder

**DOI:** 10.3389/fnins.2022.998747

**Published:** 2022-09-16

**Authors:** Shuyun Li, Xiaobing Lu, Xiaodong Chen, Zebin Huang, Hui Zhou, Zezhi Li, Yuping Ning

**Affiliations:** ^1^The First School of Clinical Medicine, Southern Medical University, Guangzhou, China; ^2^The Affiliated Brain Hospital of Guangzhou Medical University, Guangzhou, China; ^3^Guangdong Engineering Technology Research Center for Translational Medicine of Mental Disorders, Guangzhou, China

**Keywords:** bipolar disorder, hyperuricemia, metabolic syndrome, triglyceride, body mass index

## Abstract

**Objective:**

The prevalence and clinically associated factors of hyperuricemia (HUA) have been widely studied in the general population but rarely in patients with bipolar disorder (BPD) co-morbid with HUA. This study attempted to investigate the prevalence of HUA in BPD patients and analyze the associated correlates of HUA.

**Materials and methods:**

In this study, 182 outpatients with BPD and 182 healthy controls participated. The demographic and clinical information were collected. The body weight, height, waist circumference (WC), hip circumference (HC), and blood pressure (BP) were measured. The levels of serum uric acid (UA), triglyceride (TG), high-density lipoprotein (HDL-C), and fasting blood glucose (FBG) were also determined.

**Results:**

BPD patients had a significantly higher prevalence of HUA (40.7%) compared to healthy controls (30.2%) (χ^2^ = 4.335, *P* = 0.037). The systolic blood pressure (SBP), pulse pressure (PP), FBG, UA, and body mass index (BMI) were higher in the BPD group compared with those in the control group, while the diastolic blood pressure (DBP) and HDL-C level were lower (*P* < 0.05) in BPD patients. The prevalence of HUA was higher in BPD patients who used antipsychotics combined with mood stabilizers than that in BPD subjects receiving the mood stabilizers alone (*P* < 0.001). The prevalence of HUA and increased serum UA levels were higher in the manic group (62.1%) than in the depressive (34.3%) or euthymia group (17.0%) (*P* < 0.001). Additionally, the severity of mania was positively correlated with the UA level (*r* = 0.410, *P* < 0.001). There were significant differences in terms of MetS (29.7% vs. 14.8%), BMI, HC, WC, TG, and HDL-C between the HUA and the non-HUA groups (*P* < 0.05). The unconditional logistic regression analysis revealed that high BMI (OR = 1.210; 95%CI: 1.100–1.331) and high TG level (OR = 1.652; 95%CI: 1.058–2.580) were the major risk factorids for HUA in BPD patients.

**Conclusion:**

Our study suggests that patients with BPD are prone to metabolic diseases such as HUA. Higher serum levels of TG and high BMI could be associated with HUA development. Clinicians need to regularly monitor and evaluate BPD patients for their serum UA levels, especially for BPD patients with manic/hypomanic episodes and/or under the treatment of antipsychotics combined with mood stabilizers.

## Introduction

Bipolar disorder (BPD) is a severe and complex psychiatric disorder and is the 17th leading cause of psycho-social disability worldwide (Vigo et al., 2016). BPD usually manifests during adolescence between the ages of 15 and 25 (Kessing et al., 2015), with a lifetime prevalence of 2.4% of the global population (Merikangas et al., 2011). Patients exhibit severely disoriented emotional and social behaviors, leading to a decreased cognitive level, poor quality of life, and perturbed social relationships (Michalak et al., 2011). BPD is also considered one of the leading causes of mental disorder-related death in young adults (Barnett, 2018). The underlying BPD condition can increase the risk of death by two times (Walker et al., 2015), simultaneously reducing the life expectancy of patients by 10–20 years than that of the normal aging population (Miller and Bauer, 2014; Chan et al., 2021). The results of a 30-year follow-up study have indicated that circulatory disease is one of the leading causes of death in BPD individuals (Ng et al., 2015). For example, about 35–40% of deaths in BPD patients could be attributed to cardiovascular diseases (CVD) (Miller and Bauer, 2014; Correll et al., 2017). Elevated uric acid (UA) level is an independent predictor of risks of CVD (Grassi et al., 2013; Jiménez-Fernández et al., 2021; Shahin et al., 2021). A meta-analysis has found that BPD patients have significantly higher UA levels than the normal population (Bartoli et al., 2016). Additionally, Chen et al. (2019) have revealed that the UA level of the first-episode BPD patients is higher than that of the healthy age-matched controls, even after the treatment of the underlying disease. Therefore, the correlation between higher UA levels and BPD onset has attracted substantial therapeutic attention. Several researchers speculate that BPD patients may have a dysfunctional purinergic system that contributes to the abnormally high UA level in these subjects, suggesting the UA level as a potential biomarker of BPD (Kesebir et al., 2014; Teixeira et al., 2016; Bartoli et al., 2017a).

Hyperuricemia (HUA) is caused by the disordered purine metabolism system. The prevalence of HUA in Chinese adults ranges from 8.4 to 13.3%. A growing body of evidence suggests that HUA induces multi-organ failure symptoms, including kidney dysfunction, hepatic disorder, endocrinal and metabolic abnormalities, cerebrovascular, and cardiovascular disorders, as well (Multi-Disciplinary Expert Task Force on Hyperuricemia and Its Related Diseases, 2017; Shi et al., 2021). The prevalence and clinically associated factors of HUA have been widely investigated in the general population, but rarely in BPD patients co-morbid with HUA. A study has reported that the prevalence of HUA is higher in hospitalized BPD patients than that in healthy controls, suggesting that HUA might have an association with metabolic syndromes (MetSs), especially among aged patients (Chen et al., 2018). However, this study did not include outpatients with BPD in determining the rate of prevalence of HUA. The association of HUA with episodes of BPD should be analyzed in more detail. An increasing number of research findings suggest that BD patients may have dysfunctions in their purinergic systems (Kesebir et al., 2014; Bartoli et al., 2016). Therefore, the major goal of this study was to investigate the prevalence of HUA in outpatients with BPD and reveal any related clinical correlates in BPD patients co-morbid with HUA, including their socio-demographic and clinical characteristics.

## Materials and methods

### Subjects

This study included an equal number of BPD outpatients and age-matched healthy controls (*n* = 182). The Ethics Committee of the Brain Hospital affiliated with Guangzhou Medical University approved the study protocol (No. AF/SC-07/02.2). Patients, who visited the hospital outpatient clinic, were recruited between October 2019 and July 2020, after they signed written informed consent for their voluntary participation in this study.

An expert psychiatrist screened BPD patients as well as healthy controls. The included participants met the following eligibility criteria: (1) should fulfill the diagnostic criteria for BPD according to the DSM-IV-TR (for patients); (2) 18–65 years of age; (3) Han Chinese descendants; and (4) must agree to sign informed consent. While patients were excluded if they had: (1) serious physical illness or recently undergone surgery; (2) organic brain disease or dementia; (3) suffered or been suffering from psychoactive or non-addictive substance abuse; and (4) pregnancy or breastfeeding condition.

### Clinical interview and assessments

#### Data collection

Patients’ demographics included data regarding height, weight, waist circumference (WC), hip circumference (HC), blood pressure (BP), as well as smoking and drinking habits.

Measurement criteria: The height and weight were measured on a fixed weight scale in the outpatient clinic.

Body mass index (BMI) was calculated as weight (kg)/height^2^(m^2^).

WC was determined by measuring the peripheral length of the waist starting from the center of the umbilicus using a soft ruler at the end of exhalation.

HC was defined by the horizontal circumference of the most prominent part of the hip measured with a soft ruler. The patient’s BP was measured in a sitting position with a standard mercury manometer after a 10-min break. Smoking habit was considered positive if the patient smoked at least one cigarette per day for the last one month, averaging at least 5 days per week. Alcohol consumption habit was considered positive if the regular alcohol consumption rate was 1–3 times a week with at least 250 ml of beer, 50 ml of wine, or 25 ml of liquor each time for the last 1 month.

#### Blood sample collection and testing

Subjects had a light diet on the day before blood sampling and avoided intake of seafood, broth, alcohol, and other diets with high purine contents (common purine-rich foods were described in the publicity pamphlet). After fasting for 12 h, venous blood was taken between 7:00 and 8:00 a.m. on the following day. Then serum samples were tested for levels of UA, triglyceride (TG), high-density lipoprotein-cholesterol (HDL-C), and fasting blood glucose (FBG) using a fully automated biochemical analyzer (AU5800).

#### The diagnostic criteria for hyperuricemia and metabolic syndrome

The main diagnostic criterion for HUA was the UA level of > 420 μmol/L for men or > 360 μmol/L for women. The diagnostic criteria for the MetS were adopted from the National Cholesterol Education Program-Third Adult Treatment Panel (NCEP-ATP III) diagnostic guidelines, which suggested at least three risk factors: (1) obesity/moderate obesity: male WC ≥ 90 cm, female WC ≥ 80 cm; (2) hypertension: systolic BP (SBP)/diastolic BP (DBP) ≥ 130/85 mmHg or taking anti-hypertensive drugs; (3) FBG level ≥ 5.6 mmol/L; (4) TG level ≥ 1.7 mmol/L; and (5) HDL-C levels for females should be ≤ 1.29 mmol/L, and for males ≤ 1.03 mmol/L.

#### Assessment of illness episodes

Depression symptoms and severity were assessed using the Hamilton Depression scale-17 (HAMD-17). The Yong mania rating scale (YMRS) was used to evaluate manic symptoms and severity such as mania/hypomania was considered when the YMRS score was > 6 points. Depression was taken into consideration if the HAMD-17 score was > 7 points. Euthymia was diagnosed at YMRS < 6 points and HAMD-17 < 7 points. Mixed symptoms were indicated at YMRS > 6 points and HAMD-17 > 7 points.

### Statistical analysis

All the statistical data analyses were performed with EpiData 3.1 and SPSS 19.0 tools. Measurement data are expressed as the mean ± standard deviation (SD), and count data as a constituent ratio or rate. Demographic data and laboratory results were analyzed using an independent samples *t*-test or chi-squared (χ^2^)-test. The χ^2^-test was used to compare the differences in the prevalence rates of HUA among the disease subtypes and drug-treated groups. The Student–Newman–Keuls’ (SNK) *post hoc* multiple comparisons were carried out to analyze the differences in serum UA levels among the disease subtypes. The variables with *P*-values of < 0.05 in the univariate analysis were included in the unconditional logistic regression model for further analysis. A *P*-value of less than 0.05 was considered significant.

## Results

### Demographic and clinical characteristics of subjects

There were no significant differences in age, gender, educational level, smoking, and drinking habits between the BPD and the control groups (All *P* > 0.05; Table 1).

**TABLE 1:** Social demographic of BPD patients and health controls.

Variable	Patients (*n* = 182)	Health controls (*n* = 182)	*t/*χ*^2^*	*P*
Male, n (%)	64 (35.2)	73 (40.1)	0.95	0.33
Age (years)	31.06 ± 11.12	30.13 ± 10.22	0.83	0.41
Education (>12 years), n (%)	109 (59.9)	121 (66.5)	1.70	0.19
Smoking, n (%)	22 (12.1)	14 (7.7)	1.97	0.16
Drinking, n (%)	9 (4.9)	12 (6.6)	0.46	0.50

BPD, bipolar disorder.

### The prevalence of hyperuricemia, metabolic syndrome, and related clinical indicators of subjects

Rates of the prevalence of both HUA and MetS were significantly higher in BPD patients compared with that of the controls (40.7% vs. 30.2%, χ*^2^* = 4.335, *P* = 0.037; and 21.4% vs. 9.3%, χ^2^ = 10.214, *P* < 0.001, respectively). Compared with the control group, the BPD group had higher SBP (*t* = 2.683, *P* = 0.008), lower DBP (*t* = 3.459, *P* = 0.001), and larger pulse pressure (PP) (*t* = 6.472, *P* = 0.001). Also, the FBG (*t* = 2.236, *P* = 0.026), serum UA level (*t* = 2.058, *P* = 0.040), and BMI (*t* = 5.673, *P* < 0.001) were higher in BPD patients than in age-matched controls, while HDL-C levels were lower (*t* = 5.967, *P* < 0.001) in BPD patients. There was no significant difference in TG levels between the two groups (*t* = 0.037, *P* = 0.970; Table 2).

**TABLE 2:** Clinical characteristics of BPD and health controls.

Variable	Patients (*n* = 182)	Health controls (*n* = 182)	*t/*χ*^2^*	*P*
Systolic BP (mmHg)	119.02 ± 13.92	115.29 ± 12.54	2.68	0.008
Diastolic BP (mmHg)	73.49 ± 10.86	77.01 ± 8.41	3.46	0.001
PP (mmHg)	45.53 ± 9.90	38.28 ± 11.41	6.47	<0.001
TG (mmol/L)	1.44 ± 0.93	1.44 ± 0.88	0.04	0.970
HDL-C (mmol/L)	1.40 ± 0.37	1.68 ± 0.51	5.97	<0.001
FBG (mmol/L)	5.36 ± 1.39	5.10 ± 0.64	2.24	0.03
UA (μmol/L)	377.87 ± 102.15	356.04 ± 100.22	2.06	0.040
BMI (kg/m2)	24.83 ± 4.15	22.74 ± 2.74	5.67	<0.001
HUA, n (%)	74 (40.7)	55 (30.2)	4.34	0.04
MetS, n (%)	39 (21.4)	17 (9.3)	10.21	0.001

SBP, systolic-blood-pressure; DBP, diastolic-blood-pressure; PP, pulse-pressure; TG, triglyceride; HDL-C, high density lipoprotein; FBG, fasting blood glucose; UA, uric acid; BMI, body mass index; HUA, hyperuricemia; MetS, metabolic syndrome.

### Associations of hyperuricemia with illness episodes and medication use in bipolar disorder patients

There was a statistically significant difference in the prevalence rates of HUA between different illness episodes in BPD patients (χ^2^ = 24.775, *P* < 0.001). The χ^2^-test showed that the rate of prevalence of HUA for patients suffering from mania/hypomania (62.1%) was higher than that of depression (34.3%) or euthymia (17.0%) (*P* < 0.05). There were no significant differences between the other episode groups (*P* > 0.05). However, there were statistical differences in rates of prevalence of HUA among different drug groups (χ*^2^* = 11.225, *P* < 0.001). The prevalence of HUA in the BPD group treated with the combined doses of antipsychotics and mood stabilizers was higher than that of BPD patients treated with mood stabilizers alone (56.2% vs. 22.2%, *P* < 0.05), while there was no statistical difference between the other drug groups (All *P* > 0.05; Table 3).

**TABLE 3:** Socio-demographic and clinical characteristics of BPD with HUA.

Variable	Hyperuricemia (*n* = 74)	Non-hyperuricemia (*n* = 108)	*t/*χ*^2^*	*P*
Age (years)	31.42 ± 12.04	30.69 ± 10.65	0.43	0.67
Gender	−	−	0.39	0.53
Male, n (%)	28 (37.84)	36 (33.33)	−	−
Female, n (%)	46 (62.16)	72 (66.67)	−	−
Smoking, n (%)	5 (6.76)	17 (15.74)	3.34	0.07
Drinking, n (%)	5 (6.76)	4 (3.70)	0.34	0.56
Illness duration (month)	67.16 ± 54.14	65.30 ± 57.10	0.22	0.83
Illness episode			24.78	<0.001
Manic/hypomanic, n (%)	41 (62.1%)	25 (37.9%)		
Euthymic, n (%)	8 (17.0%0	39 (83.0%)		
Depressive, n (%)	23(34.3%)	44 (65.7%)		
Mixed, n (%)	1 (50.0%)	1 (50.0%)		
Drug use			14.20	0.01
Antipsychotics, n (%)	2 (28.6%)	5 (71.4%)		
Antipsychotics and Mood stabilizer, n (%)	41 (56.2%)	32 (43.8%)		
Mood stabilizer, n (%)	8 (22.2%)	28 (77.8%)		
Antipsychotics and antidepressants (*n* = 21)	6 (28.6%)	15 (71.4%)		
Antipsychotics and Antidepressan and Mood stabilizer (*n* = 29)	11 (37.9%)	18 (62.1%)		
Antidepressan and Mood stabilizer (*n* = 16)	6 (37.5%)	10 (62.5%)		

### Comparison of uric acid levels among different illness episodes in bipolar disorder patients

After *post doc* multiple comparisons, statistically significant differences (*P* < 0.001) were observed in UA levels between the manic (425.4 ± 102.8), the euthymia (335.3 ± 70.2), and the depressive (361.9 ± 110.9) groups, while the differences between other groups were not statistically significant (All *P* > 0.05). The severity of mania was positively correlated with the increase in the UA level (*r* = 0.410, *P* < 0.001). There was no correlation between depression and a higher UA level (*r* = −0.018, *P* = 0.813).

### Univariate analysis for differences in clinical characteristics between the hyperuricemia and non-hyperuricemia bipolar disorder patients

Compared with the non-HUA group, the HUA group exhibited a higher prevalence of MetS (29.7% vs. 14.8%, χ^2^ = 5.913, *P* = 0.015), higher BMI (*t* = 5.295, *P* < 0.001), larger HC (*t* = 5.4915, *P* < 0.001), larger WC (*t* = 5.250, *P* < 0.001), higher TG level (*t* = 4.006, *P* < 0.001), and lower HDL-C level (*t* = 2.271, *P* = 0.024). There were no significant differences in the SBP (*t* = 0.587, *P* = 0.558), DBP (*t* = 0.169, *P* = 0.866), and FBG level (*t* = 0.522, *P* = 0.602) between these two groups (All *P* > 0.05; Table 4).

**TABLE 4:** Comparison of clinical characteristics in HUA with Non-HUA in patients with BPD.

Variable	Hyperuricemia (*n* = 74)	Non-hyperuricemia (*n* = 108)	*t/*χ*^2^*	*P*
BMI (kg/m^2^)	26.67 ± 4.16	23.57 ± 3.66	5.30	<0.001
HC (cm)	99.97 ± 6.60	94.66 ± 6.29	5.49	<0.001
WC (cm)	89.68 ± 11.48	81.29 ± 9.93	5.25	<0.001
SBP (mmHg)	118.28 ± 14.78	119.52 ± 13.34	0.59	0.56
DBP (mmHg)	73.32 ± 12.13	73.60 ± 9.96	0.17	0.87
PP (mmHg)	44.94 ± 8.22	45.92 ± 10.92	0.64	0.52
TG (mmol/L)	1.76 ± 1.17	1.22 ± 0.65	4.01	<0.001
HDL-C (mmol/L)	1.32 ± 0.31	1.45 ± 0.39	2.27	0.02
FBG (mmol/L)	5.42 ± 1.41	5.31 ± 1.54	0.52	0.60
MetS, n (%)	22 (29.7%)	16 (14.8%)	5.91	0.02

HC, hip circumference; WC, waist circumference.

### Analysis of factors related to hyperuricemia in bipolar disorder patients

Having HUA as the dependent variable (HUA = 1, non-HUA = 0), the results with significant differences obtained by the univariate analysis, including the WC, HC, BMI, TG, illness episodes, and use of antipsychotics, were incorporated into the logistic regression model. As shown in Table 5, BMI and TG levels were related to the development of HUA (OR = 1.210, *P* < 0.0001; and OR = 1.652, *P* = 0.027, respectively).

**TABLE 5:** Analysis of factors related to HUA in BPD patients.

Variable	*B*	Wald χ^2^	*OR*	95%CI	*P*
BMI	0.19	15.29	1.21	1.10–1.33	<0.001
TG	0.50	4.88	1.65	1.06–2.58	0.03

## Discussion

This study aimed to investigate the prevalence of HUA in outpatients with BPD and analyze the effects of gender, age, smoking, drinking, illness duration, illness episode, use of antipsychotics, MetS, and its components on the HUA pathogenesis. The results showed that (1) BPD patients had a significantly higher prevalence of HUA than age-matched healthy controls; (2) higher prevalence of HUA and increased serum UA level were associated with manic episodes; (3) the severity of mania was positively correlated with serum UA levels of BPD patients; and (4) higher BMI and TG levels could be the major risk factors for HUA in BPD patients.

In this study, the prevalence of HUA in BPD patients was 40.7%, which was higher than that of healthy controls (30.2%). Higher prevalence of HUA and increased UA level were more closely associated with the manic episode symptoms than the depressive or euthymia episodes. The severity of mania (YMRS ratings) was positively correlated with the UA level (*r* = 0.410, *P* < 0.001). The final product of purine metabolism in humans is UA. Under normal circumstances, the production and excretion of UA are balanced. However, disordered purine metabolism can induce increased UA production and/or decreased UA excretion, leading to the development of HUA in humans. Patients with BPD may have disturbances in purine metabolism (Ortiz et al., 2015; Bartoli et al., 2017b; Malewska-Kasprzak et al., 2019). Increased UA levels, suggesting an increased purinergic transformation, have been observed in drug-naïve subjects with BPD during the first manic episode (Salvadore et al., 2010). Furthermore, these levels were positively correlated with the symptom severity in manic episodes (De Berardis et al., 2008; Machado-Vieira, 2012). In a study by Kesebir et al. (2014), a positive correlation between hyperthymic and irritable temperament scores and the UA level has been demonstrated in BPD patients. It has also been implicated in the pathophysiology and therapeutics of mood disorders, particularly for impulsivity and excitement-seeking behaviors (Machado-Vieira et al., 2002; Sutin et al., 2014). The clinical history of lithium began to surface in the mid-nineteenth century when it was used to treat gout. Lithium directly targets the adenosinergic system. Lithium inhibits the ectonucleotidase activity and the accumulation of adenosine in cells (Oliveira Rda et al., 2011; Oruch et al., 2014). One study suggests that pharmacologic agents that are capable of penetrating the blood-brain barrier (BBB) can alter the adenosine metabolism, leading to a decreased serum UA level. Effenol is a xanthine oxidase (XO) inhibitor that suppresses purine degradation, resulting in increased adenosine levels (Pacher et al., 2006). The clinical potential of the anti-gout agent allopurinol in acute bipolar mania has been recognized (Machado-Vieira et al., 2008).

We found that the prevalence of MetS was higher in BPD patients compared to that in healthy controls (21.4% vs. 9.4%). The SBP, PP, FBG, UA, and BMI in the BPD group were higher than those in the control group, while the DBP and HDL-C values in the patient group were lower than those in the control group (All *P* < 0.05). A review by Grover et al. (2012) has found MetS as a frequently occurring symptom in BPD patients, with a prevalence rate of 16.7–67% depending on different diagnostic criteria. It is worth noting that BPD patients presented higher SBP and lower DBP, along with a significantly higher PP than that of healthy controls (*P* < 0.001). The PP difference is an indicator of arteriosclerosis, especially for large arteries (Said et al., 2018), and increased PP is an independent predictor of CVD and mortality outcomes (Glasser et al., 2014), which need further attention.

Simultaneously, this study revealed a higher prevalence (29.7%) of MetS in BPD patients in the HUA group than that in controls (14.8%), indicating that the HUA might associate with the metabolic disorders in BPD patients, which was consistent with previous findings (Chen et al., 2018). Other investigations have also suggested that HUA could be a strong and independent predictor of MetS (Borges et al., 2010). Moreover, a meta-analysis of 11 prospective studies indicates an independent and linear dose-dependent relationship between HUA and MetS (Biradar et al., 2020). The HUA is closely associated with the MetS and its components (Borges et al., 2010; Thottam et al., 2017; Zhang et al., 2018; Xu et al., 2021). Here, we showed by the univariate analysis that the BMI, HC, WC, and TG were higher in the HUA subjects than those in the normal individuals, while the HDL-C level, a protective factor for CVD risk, was lower in the HUA group than in the control group. Most of these indicators were components of the MetS. The results of the multivariate analysis further suggest that both high BMI and high TG levels could be critical risk factors for HUA pathogenesis in BPD patients. BPD shares some common symptoms with obesity, such as impulsivity, cognitive impairment, overeating, and sleep disturbance (Delgado-Rico et al., 2012). Compared with the age-matched general population, BPD patients are more likely to be overweight or obese (Zhao et al., 2016), particularly with abdominal obesity (McElroy et al., 2004). After treatment, up to 68% of patients develop overweight or obesity (De Hert et al., 2011), with elevated TG levels and decreased HDL-C levels (Henderson et al., 2000). The strong affinity of antipsychotics for 5-HT2C and H1 receptors is considered the major cause of weight gain (Reynolds and McGowan, 2017). Besides, high BMI is associated with higher insulin resistance and leptin production (Yamada et al., 2011; Lubkowska et al., 2015), both of which could significantly impact the UA secretion system, resulting in an elevated level of UA. The pathological connection between high TG level and HUA onset may be related to the free fatty acid metabolism pathway. The increase in serum TG level can cause more free fatty acid production and utilization, and accelerate the decomposition of adenosine triphosphate (ATP), thereby enhancing the production of UA, the final product of purine metabolism in humans (Balasubramanian, 2003).

We could not find any statistical differences in terms of gender composition, age, educational level, smoking, and drinking status between BPD outpatients and healthy controls. According to previous studies, BPD patients have higher rates of smoking (Diaz et al., 2009) and drinking habits (Liberopoulos et al., 2004) than the general population, but this difference could be detected in this study. This might be attributed to the fact that we collected information about the smoking and drinking status of patients for the last 1 month, so that when patients were diagnosed and started receiving treatment, they could be supported with relevant health education, including rehabilitation guidance to quit smoking and drinking, which might have reduced the number of smoking and drinking cases in this study. The low sample size could also result in insufficient statistical power to reveal the relationship between these behavioral characteristics, MetS, and UA levels. Therefore, if doctors can provide health education to patients in the early stages of treatment, they may be able to change their poor lifestyle habits, thus reducing the chances of negative impact on their health.

This study has several limitations that need to be considered while interpreting the results. First, as a cross-sectional study, a causal relationship between HUA and BPD could not be inferred from the obtained results. Second, we found that the prevalence of HUA was higher in the combined antipsychotics and mood stabilizers use group than that in the mood stabilizers alone group. It is suggested that there may be a certain relationship between the use of antipsychotics and HUA pathogenesis. But drug compositions were not thoroughly analyzed to determine whether there were differences in drug types and doses between the HUA and non-HUA treatment groups. The pharmacological mechanism of each drug varies considerably from the other; hence, these drugs may have different effects on the patient’s UA level. There are no known studies of the UA level in BPD patients taking antipsychotics. And third, there were no data related to the low-density lipoprotein-cholesterol level as an indicator related to the upregulation of TG level.

In conclusion, the results of this study suggest that BPD patients are prone to developing HUA, MetS, and other metabolic diseases, as well. HUA may be associated with BPD illness episodes, the use of antipsychotics, and the underlying MetS, along with its associated pathological components. High BMI and TG levels could be the major risk factors for HUA onset. When treating BPD, clinicians need to regularly monitor the patient’s metabolic status, identify and intervene in any metabolic diseases such as HUA, and conduct appropriate health education, including guidance to quit smoking and drinking, control diet, perform regular exercise, strengthen weight management, and to pay attention to body weight and blood lipids. It is necessary to actively prevent various chronic diseases related to HUA, which in turn can reduce the adverse consequences caused by MetS, improve the prognosis, and reduce the risk of death of the patient.

## Data availability statement

The raw data supporting the conclusions of this article will be made available by the authors, without undue reservation.

## Ethics statement

The studies involving human participants were reviewed and approved by the Ethics Committee of the Brain Hospital Affiliated to Guangzhou Medical University approved this study protocol (No. AF/SC-07/02.2). The patients/participants provided their written informed consent to participate in this study.

## Author contributions

XL and YN designed the study. SL and ZL wrote the manuscript. XC, ZH, and HZ collected the data. SL did the statistical analysis. ZL and YN reviewed and revised the manuscript. All authors contributed to the article and approved the submitted version.
